# Ataxia–Telangiectasia and Associated Bronchiectasis: Case Report and Literature Review

**DOI:** 10.3390/jcm15124524

**Published:** 2026-06-11

**Authors:** Roxana Taraș, Marina Dima, Mihaela Axente, Eliza Elena Cinteză, Cherecheș-Panța Paraschiva, Claudia Lucia Toma, Ruxandra Vidlescu, Marcela Daniela Ionescu

**Affiliations:** 1 Department of Pediatrics, Carol Davila University of Medicine and Pharmacy, 020021 Bucharest, Romania; roxana.taras@umfcd.ro (R.T.); eliza.cinteza@umfcd.ro (E.E.C.); daniela.ionescu@umfcd.ro (M.D.I.); 2 Department of Pediatrics, “M.S. Curie” Emergency Clinical Hospital for Children, 077120 Bucharest, Romania; marina.dima1511@gmail.com; 3 Department of Neonatology, “M.S. Curie” Emergency Clinical Hospital for Children, 077120 Bucharest, Romania; mihaela.axente@gmail.com; 4 Department of Pediatric Cardiology, “M.S. Curie” Emergency Clinical Hospital for Children, 077120 Bucharest, Romania; 5 Department of Mother and Child, Discipline Pediatric III, University of Medicine and Pharmacy “Iuliu Hațieganu”, 400012 Cluj Napoca, Romania; pusachereches@umfcluj.ro; 6 Department of Pediatrics, “Children’s Emergency Clinical Hospital”, 400012 Cluj-Napoca, Romania; 7 Department of Pulmonology, Carol Davila University of Medicine and Pharmacy, 020021 Bucharest, Romania; claudia.toma@umfcd.ro; 8 Marius Nasta Institute of Pulmonology, 050159 Bucharest, Romania; 9 Department of Pediatric Oncology, “M.S. Curie” Emergency Clinical Hospital for Children, 077120 Bucharest, Romania

**Keywords:** bronchiectasis, ataxia–telangiectasia, immunodeficiency

## Abstract

Ataxia–telangiectasia is a rare, autosomal recessive primary immunodeficiency caused by mutations in the *ATM* gene on chromosome 11, which encodes a serine–threonine kinase essential for the recognition and repair of DNA double-strand breaks. The disease is characterized by progressive neurological impairment, immunological dysfunction, and an increased susceptibility to recurrent infections and malignancies. Pulmonary involvement represents a major source of morbidity and frequently arises from chronic infections, aspiration, and impaired airway clearance, ultimately leading to the development of bronchiectasis. The case of a 15-year-old adolescent with a history of recurrent aspiration pneumonias, neuropsychomotor developmental delay, and severe malnutrition is reported, who was admitted for evaluation of chronic productive cough, fever, and dysphagia. Comprehensive clinical assessment and ancillary investigations revealed recurrent respiratory infections, gastroesophageal reflux, and typical features of ataxia–telangiectasia, including cerebellar ataxia, oculomotor apraxia, and conjunctival telangiectasias. Additionally, bronchiectasis was identified as a secondary consequence of the underlying neurological and immunological impairment. This case highlights the diagnostic challenges posed by ataxia–telangiectasia in pediatric patients presenting with chronic respiratory symptoms and emphasizes the importance of early recognition of the underlying systemic disorder. A multidisciplinary approach is essential for accurate diagnosis and optimized management, aiming to address both the primary disease and its pulmonary complications.

## 1. Introduction

Ataxia–telangiectasia (A–T) is a rare autosomal recessive primary immunodeficiency caused by mutations in the ATM gene, leading to progressive cerebellar dysfunction and immunodeficiency. Patients with this disease are highly susceptible to recurrent sinopulmonary infections, chronic lung disease, and aspiration-related complications, which frequently lead to bronchiectasis and constitute major contributors to morbidity and reduced life expectancy. Respiratory disease accounts for approximately one third of deaths in patients with ataxia–telangiectasia and represents one of the leading causes of mortality alongside malignancy [1,2].

The case report presented below describes a 15-year-old adolescent with recurrent aspiration pneumonias, neurodevelopmental delay, and severe malnutrition admitted for chronic productive cough, fever, and dysphagia. Clinical evaluation revealed characteristic features of ataxia–telangiectasia, including cerebellar ataxia, oculomotor apraxia, and conjunctival telangiectasias, along with gastroesophageal reflux. Pulmonary imaging identified bronchiectasis, reflecting secondary complications of the underlying neurological and immunological deficits.

This case highlights the intricate relationship between systemic and respiratory manifestations in ataxia–telangiectasia, with aspiration-related disease playing a central role in recurrent pneumonia and structural lung damage. It further illustrates how overlapping neurological and respiratory features can complicate and delay diagnosis, while the early and severe development of bronchiectasis underscores the rapid progression of pulmonary involvement and the need for timely multidisciplinary evaluation. The present case is consistent with previously reported studies describing respiratory complications as a major source of morbidity in ataxia–telangiectasia. However, in contrast to many reported cases in which infectious and immunological factors predominate, the current patient exhibited a marked contribution of aspiration-related lung disease secondary to significant neurodevelopmental impairment and dysphagia. This emphasizes the role of neurological dysfunction as a critical, and sometimes underrecognized, driver of chronic pulmonary damage in A–T. In addition, bronchiectasis in patients with A–T is generally described as a progressive complication developing over time in the context of recurrent infections. In this case, however, severe structural lung disease was identified at a relatively young age, suggesting an accelerated pulmonary decline likely influenced by repeated aspiration events and severe malnutrition. This pattern may indicate a more aggressive respiratory phenotype characterized by combined infectious, neurological, and nutritional contributors.

The aim of this study was to report the case of an adolescent with ataxia–telangiectasia complicated by bronchiectasis and recurrent aspiration pneumonia, emphasizing the diagnostic challenges and pulmonary manifestations of this rare multisystem disorder, and to provide a concise review of the current literature regarding respiratory involvement, multidisciplinary management, and clinical outcomes in patients with A–T.

## 2. Case Presentation

A 15-year-old male adolescent presented to the hospital with fever; persistent, paroxysmal wet cough; and dysphagia. Symptoms began approximately three months earlier, initially with dysphagia for solids and subsequently for liquids, alongside a wet cough; over the last ~10 days he developed fever and respiratory difficulty.

Past medical history included multiple hospitalizations for protracted, recurrent aspiration pneumonias and several chronic conditions: global developmental disorder, chronic infantile encephalopathy, grade III protein–calorie malnutrition, spastic tetraparesis, bilateral hydronephrosis, intellectual disability, and adductor coxa deformity for which surgery was performed approximately two years prior.

Family history revealed maternal-line oncologic disease with fatal outcomes: the maternal grandmother died at 63 years, and two maternal aunts at 32 and 40 years, all with breast cancer. The 38-year-old mother is reportedly healthy. The 57-year-old father is also reportedly healthy, with no known paternal-line history of oncologic, genetic, or immunologic conditions or premature deaths. The patient has three clinically healthy sisters aged 21, 18, and 13 years, and two brothers—one died at age 7 from acute myeloblastic leukemia while undergoing chemotherapy, and the other, aged 11, exhibits a clinical picture suggestive of ataxia–telangiectasia, with neuropsychomotor developmental delay (independent ambulation achieved at 3 years), speech disorder with unclear diction and articulation difficulties, ataxia, conjunctival telangiectasias, and visual disturbances.

Perinatal history involved an uncomplicated pregnancy, being the fourth birth after three term deliveries and one post-traumatic abortion. Delivery was spontaneous at term; birthweight was 4.100 g. The newborn had physiologic jaundice requiring phototherapy. Postnatal adaptation was favorable. Vaccination was carried out following the national schedule.

Physical examination on admission showed an overall condition relatively satisfactory given the underlying disease, but with evident nutritional and neuro-psychomotor impairment. Weight was 26 kg (*p* = 0.1%, Z-score −5.59), indicating severe underweight, and head circumference was 51 cm (−3.3 SD), suggestive of microcephaly. Phenotype was notable for dysmorphic facial features, exophthalmos, hypotrophy, and cachectic trunk. Skin was pale; during coughing fits, bilateral supraclavicular petechiae were observed, with telangiectatic-appearing lesions between coughing episodes. Ocular findings included horizontal nystagmus on lateral gaze bilaterally, oculomotor apraxia, conjunctival vessel dilatation and hyperemia more pronounced during coughing, supporting suspicion of telangiectatic vascular changes. Hair and nails appeared normal; subcutaneous adipose tissue was poorly represented. No peripheral lymphadenopathy was detected. Neuromuscularly, there was a spastic–dyskinetic pattern with axial hypotonia and muscle hypotrophy in a patient with spastic tetraparesis. Lower limbs were fixed in flexion; gait was ataxic with a broad-based stance. The patient could not maintain independent orthostatism, ambulated with bilateral support, and took a few steps with the knees flexed. There was no truncal ataxia. Fine motor function was markedly impaired, with fine intention tremor and dystonic posturing of the fingers of the upper limbs, requiring assistance for basic activities. The musculoskeletal system had an apparently normal conformation.

Respiratory assessment: Oxygen saturation was 94% on room air and 96% on oxygen via face mask at 4 L/min; paroxysmal spastic cough was observed with no cyanosis. Chest auscultation: Vesicular breath sounds were present bilaterally and symmetrically, mildly diminished over the right hemithorax, with additional fine crackles, scattered rhonchi, and basal coarse crackles in both lung fields.

Cardiac examination: Regular rhythm was observed, with a heart rate of 108 bpm; heart sounds were rhythmic, well perceived, and had no pathological murmurs. Appetite was capricious; pharyngeal inspection revealed moderate hyperemia. Abdomen had normal contour and was soft, mobile with respiration, and non-tender spontaneously and to palpation. Non-tender hepatomegaly was palpable, with the superior hepatic border at the right sixth intercostal space. No splenomegaly was detected on physical examination. Urinary function was preserved with present diuresis and normochromic urine. External genitalia was normally developed and sex-concordant.

Neurologically, there was global neuropsychomotor developmental delay; alertness and spatiotemporal orientation were present; finger-to-nose testing showed tremor and dysmetria; and axial ataxia was observed (unable to sit unsupported). No meningeal irritation signs were observed. Cognitively, the patient could count to five with assistance, did not correctly recognize colors, could state his own and family members’ names and his age, used a phone (knew how to call and show photos), knew his location, answered coherently, and cooperated with the examiner; dysarthria was noted.

Laboratory findings showed leukocytosis (26,600/µL) with neutrophilia (20,350/µL), moderate hypochromic microcytic anemia (Hb 8.8 g/dL) with low serum iron and elevated ferritin in an inflammatory context, thrombocytosis likely infection-related (734,000/µL), inflammatory syndrome with C-reactive protein ~10× the upper normal limit, normal procalcitonin, elevated LDH (307 U/L), negative Quantiferon-TB Gold, negative serology for *Mycoplasma pneumoniae*, and arterial blood gas values near normal; additionally AST, ALT, CK, CK-MB, urea, and creatinine levels were within normal limits, and urinalysis was negative.

**Figure 1:**
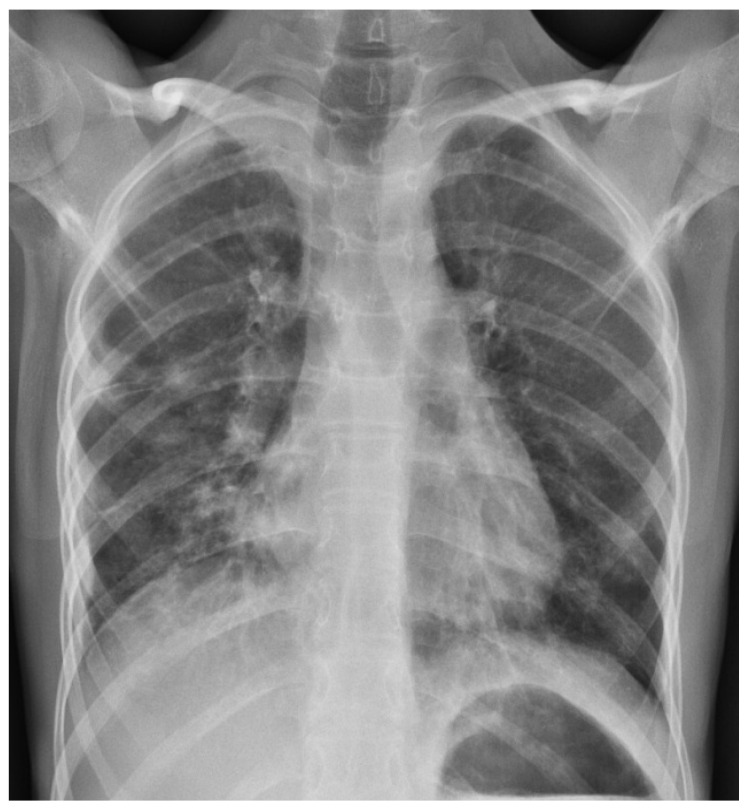
Anteroposterior (AP) chest radiograph of a 15-year-old adolescent with recurrent aspiration pneumonia. Alveolar consolidation foci are observed in the left retrocardiac area, as well as right infrahilar alveolar infiltrates, a rounded right lateral costophrenic sinus, and a sharp left lateral costophrenic sinus.

Chest radiograph revealed retrocardiac left alveolar consolidations, right infrahilar alveolar infiltrates, a rounded right lateral costophrenic sinus, and a sharp left lateral costophrenic sinus, as presented in Figure 1.

Abdominal ultrasound showed no significant abnormalities. The liver had normal dimensions (right hepatic lobe “prerenal” diameter = 12.7 cm; left hepatic lobe anteroposterior diameter = 4.4 cm), smooth contour, homogeneous echotexture, no sonographically visible focal lesions, no intra- or extrahepatic bile duct dilatation, and a normal-appearing hepatic vascular tree. The gallbladder had thin walls with anechoic content, without images suggestive of calculi. The pancreas was visualized caudally, with normal size, homogeneous, normoechoic echotexture, and no focal lesions. The spleen measured within normal limits (longitudinal axis = 9.8 cm) with homogeneous echotexture and sharp contours. Both kidneys were in normal position and showed normal size (right kidney = 9/3.8/3.7 cm; left kidney = 9/4.7/4.6 cm), with normal parenchymal thickness, regular contours, and good corticomedullary differentiation; no pelvicalyceal dilatation or calculi were identified. The ureters were not visualized proximally or distally, and the urinary bladder was non-assessable due to diffuse bowel gas (aerocolia).

**Figure 2:**
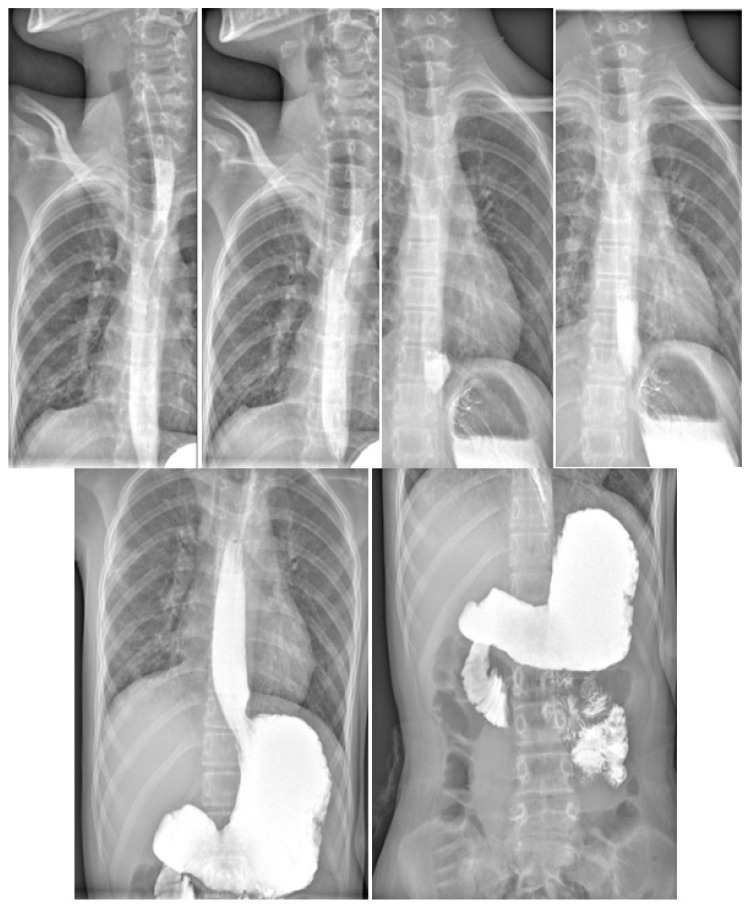
Barium swallow showing the presence of gastroesophageal reflux in a 15-year-old adolescent with recurrent aspiration pneumonia.

Given the symptomatology and the history of recurrent aspiration pneumonias, a barium transit study was considered to objectify a possible foregut malformation or the presence of gastroesophageal reflux. As illustrated in Figure 2, barium study performed in lateral decubitus and in the Trendelenburg position demonstrated retrograde passage of contrast with opacification of the entire esophagus following gastric filling. In the supine position, administration of contrast showed an esophagus with normal course and caliber; without dilatations or stenotic segments; with smooth, regular contours; and good antegrade transit of barium across the cardia, which was located subdiaphragmatically. The stomach was normally positioned, normotonic, normokinetic, and normosecretory, with well-defined gastric surfaces and curvatures and flattening of the gastric folds at the antrum. There was good emptying through a central pylorus, a normally opacified triangular duodenal bulb, a normally configured duodenal loop with mild hypotonia, and intestinal loops with normal topography and no caliber abnormalities.

Given the history of multiple protracted aspiration pneumonias and the symptomatology, clinical examination, laboratory data, and imaging findings, intravenous antibiotic therapy with meropenem and teicoplanin was initiated for 11 days. Under this regimen, the patient’s evolution was favorable, with improvement of symptoms, resolution of the inflammatory syndrome, and normalization of the complete blood count.

The patient’s recurrent respiratory pathology appears to have a multifactorial mechanism requiring a multidisciplinary approach for further evaluation.

On the one hand, the repeated episodes of aspiration pneumonia are attributable to impaired coordination of swallowing and cough reflex, as well as gastroesophageal reflux demonstrated on the barium swallow study.

On the other hand, based on the clinical findings, features suggestive of an underlying neurological disorder contributing to recurrent aspiration were also identified. Neurological examination revealed an ataxic syndrome, severe tendon contractures, an atypical pyramidal–extrapyramidal pattern with absent deep tendon reflexes and no clonus, oculomotor apraxia, and telangiectatic vascular abnormalities at the conjunctival, cervical, and supraclavicular regions. The combination of these signs—particularly the triad of ataxia, oculomotor apraxia, and telangiectasias—resulted in a high clinical suspicion of ataxia–telangiectasia (Louis-Bar syndrome). To confirm the diagnosis and assess the extent of multisystem involvement over time, additional imaging and paraclinical investigations were subsequently performed.

**Figure 3:**
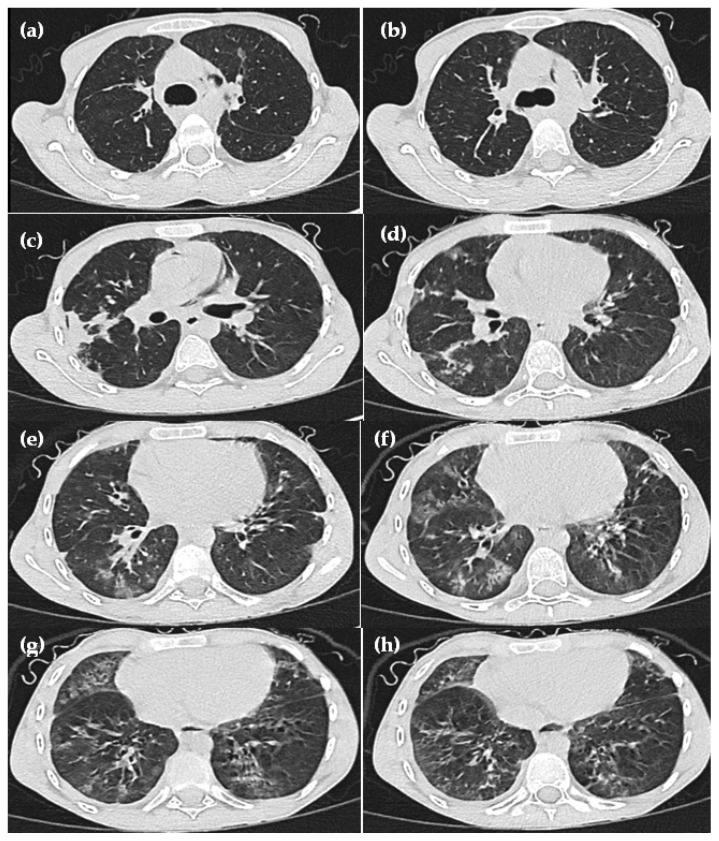
Chest computed tomography (CT) in an adolescent with ataxia–telangiectasia demonstrating multiple cylindrical bronchiectases in both lung fields (**a**–**h**), small diffuse ground-glass opacities and pseudonodular alveolar consolidations, predominantly peripheral and basal, mainly in the right lung (**c**,**d**). A “tree-in-bud” pattern is seen in the right lower lobe (**f**–**h**).

Chest computed tomography revealed an enlarged trachea cranial to the carina (antero-posterior diameter = 21 mm; transverse diameter = 18 mm), with a dependent hypodense deposit in the distal trachea and along the right main bronchus (over ~20 mm from its origin). As evidenced in Figure 3, multiple cylindrical bronchiectasis were noted, most with patent lumens, with possible impaction in the bronchus of the posterior segment of the right upper lobe; distally, peripherally, and subpleurally, a zone of consolidation ± atelectasis was observed. At the right apex, multiple confluent air cysts up to 6.5 mm were evident, with interstitial thickening at this level, as well as a 6 mm intraparenchymal air cyst in the left upper lobe. Scattered ground-glass opacities and pseudo-nodular alveolar consolidations were seen, mainly peripherally and basally, predominating in the right lung. A “tree-in-bud” appearance was identified in the right lower lobe, as presented in Figure 3f–h. In addition, focal pleural thickening was observed in the laterobasal region of the left lower lobe. CT also demonstrated a few mediastinal lymph nodes, the largest infracarinal (11.5 × 6 mm). A pneumomediastinum was identified by the presence of fine air lines outlining the pulmonary artery, also visible in the aortopulmonary window. No pleural or pericardial effusions were detected, and no air was present in the pleural cavity.

**Figure 4:**
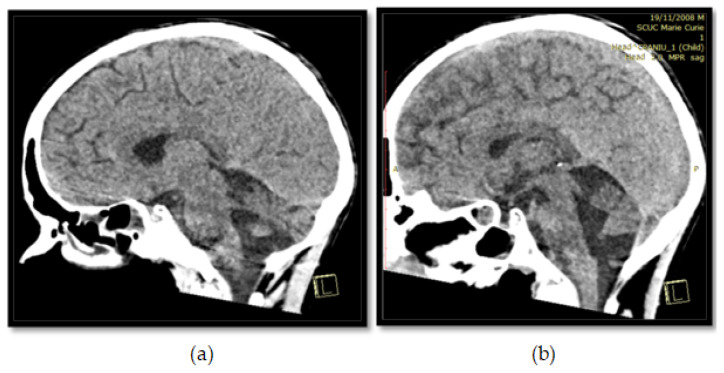
CT scan of a 15-year-old male adolescent with ataxia–telangiectasia, demonstrating the presence of hypoplasia of the cerebellum and hypoplasia of the brainstem (**a**,**b**).

Brain computed tomography revealed paranasal sinusitis as well as hypoplasia of the cerebellum and brainstem, as illustrated in Figure 4. Hypodense content was also noted within the sphenoidal and maxillary sinuses and in several ethmoidal air cells. In the posterior cranial fossa, there was widening of the pericerebellar space (cisterna magna and ambient cistern), enlargement of the fourth ventricle, and deepening of the cerebellar cortical sulci. Supratentorially, the pericerebral space and interhemispheric fissure were of normal dimensions; cortical relief was well represented, with preserved cortico-subcortical differentiation. The ventricular system was midline and symmetric, with the lateral ventricles and third ventricle of normal size; the basal ganglia displayed a normal tomographic appearance. No spontaneous hyper- or hypodense lesions were detected in the cerebral or cerebellar parenchyma.

Extensive laboratory investigations revealed elevated alpha-fetoprotein (745 ng/mL; upper limit 12 ng/mL) and an immunogram showing decreased IgA and IgG levels, findings that support the presumptive diagnosis.

Given the strong clinical suspicion of ataxia–telangiectasia (Louis-Bar syndrome), targeted genetic testing was performed for mutations in the *ATM* gene to confirm the diagnosis. Two heterozygous variants were identified in *ATM* (NM_000051.4): c.5979_5983del (p.Ser1993Argfs23), a frameshift variant classified as pathogenic, and c.5075_5076del (p.Lys1692Argfs9), a frameshift variant classified as probably pathogenic. Both are associated with disorders within the ATM-related spectrum. These results support the diagnosis of ataxia–telangiectasia in the described clinical context.

Based on these findings, the patient was deemed eligible for replacement therapy with intravenous immunoglobulins, with an initial dose of 0.5–1 g/kg per infusion, as long-term treatment under the National Program for Primary Immunodeficiencies.

## 3. Literature Review: Ataxia–Telangiectasia and Associated Bronchiectasis

Ataxia–telangiectasia (A–T) is a rare, autosomal recessive multisystem disorder characterized by progressive cerebellar ataxia, oculomotor apraxia, immunodeficiency, and vascular telangiectasias. Pulmonary involvement is a major cause of morbidity in A–T, with recurrent respiratory infections, impaired airway clearance, and aspiration contributing to the development of bronchiectasis [1,2].

Depending on etiology, bronchiectasis can be classified into two main categories: cystic fibrosis bronchiectasis (CFB) and non-cystic fibrosis bronchiectasis (NCFB) [3]. In the context of A–T, bronchiectasis represents a form of NCFB arising secondary to chronic infections and the underlying neurological and immunological dysfunction [4,5].

### 3.1. Ataxia–Telangiectasia—A Rare Cause of NCFB

Ataxia–telangiectasia is a rare, autosomal recessive primary immunodeficiency caused by mutations in the *ATM* gene on chromosome 11, which encodes a serine–threonine kinase responsible for recognizing and repairing DNA double-strand breaks [1,2]. Its estimated global prevalence is 1 in 40,000–100,000 live births; it is more frequent in the Caucasian population and affects both sexes approximately equally [6,7]. In the United States, roughly 1% of the population are carriers of an *ATM* mutation. This complex disorder manifests in the first decade of life [1].

Neurological symptoms (ataxia and axonal polyneuropathy) often begin in early childhood, with ataxia frequently being the first alarming sign for parents [6]. Subsequently, mucocutaneous and visceral telangiectasias (brain and urinary bladder) appear; ocular telangiectasias are pathognomonic [8,9]. As the disease progresses, growth and developmental delay, oculomotor apraxia, nystagmus, and dysphagia are observed. Basal ganglia involvement leads to parkinsonian tremor, chorea, dystonia, and myoclonus [1]. Mild-to-moderate cognitive deficits have also been reported, affecting language, memory, and executive function [10].

Beyond neurological manifestations, patients may exhibit additional conditions: immunologic (immunoglobulin, antibody deficiencies, and lymphopenia), endocrine (insulin resistance and diabetes mellitus), dermatologic, and pulmonary (recurrent infections) conditions and a heightened susceptibility to malignancy [8,9,10,11]. Approximately 25–30% of individuals with ataxia–telangiectasia develop neoplastic diseases—most commonly leukemia and lymphoma—at a young age [1]. Tumors may be accompanied by marked radiosensitivity. Evidence from the literature shows that X-rays and gamma radiation are particularly harmful in ataxia–telangiectasia and should be avoided, in contrast to ultraviolet radiation, which has been used in the management of malignancies in this patient population [12].

Three major phenotypes of pulmonary disease have been recognized in patients with ataxia–telangiectasia: (1) recurrent upper and lower respiratory tract infections (RTIs), which can subsequently lead to bronchiectasis; (2) pulmonary disease associated with neuromuscular deficits affecting the bulbar and spinal systems, resulting in dysfunctional swallowing and ineffective cough, thereby increasing the risk of aspiration and potentially causing or exacerbating bronchiectasis; and (3) interstitial lung disease/pulmonary fibrosis. Although there is often substantial overlap among these phenotypes, they will be presented separately below [2,4].

Recurrent sinopulmonary infections represent a significant clinical concern in individuals with A–T and are largely attributable to selective IgA deficiency, hypogammaglobulinemia, impaired antibody production—particularly against polysaccharide antigens—IgG subclass deficiencies, and T-cell lymphopenia and dysfunction. Recent studies have shown that patients with mutations causing complete loss of ATM kinase activity are more likely to experience recurrent respiratory infections and need prophylactic antibiotics than those with mutations that allow some residual kinase activity. This suggests that even a small amount of ATM function helps protect against immune deficiency. In a large French registry, Micol et al. classified ATM mutations as either complete loss of expression or activity (class A, null) or partial/reduced activity (class B, hypomorphic). They found that respiratory infections (RTIs) were the main risk factor for mortality in patients with hypomorphic ATM mutations, whereas those with null mutations had a higher risk of developing cancer (mainly hematologic malignancies) at younger ages. In addition to these immune defects, chronic inflammation, impaired pulmonary clearance, and recurrent aspiration likely contribute to the recurrent and chronic pulmonary infections observed in A–T [4,5].

In ataxia–telangiectasia (A–T), pulmonary complications frequently arise from progressive neuromuscular dysfunction. Bulbar and respiratory muscle impairment leads to dysphagia, chronic aspiration, reduced tidal volumes, and ineffective cough, promoting recurrent infections and progressive lung injury. The consequent accumulation of secretions and impaired airway clearance further exacerbates respiratory morbidity, contributing to conditions such as bronchiectasis and restrictive lung disease [2,4].

Interstitial lung disease (ILD) has been described in individuals with ataxia–telangiectasia, although its exact incidence remains unknown. Schroeder and colleagues conducted a retrospective study including 437 patients with A–T and identified 25 patients diagnosed with ILD, with a mean age of onset of 17.5 years. Non-productive cough, dyspnea, and fever were the most common symptoms preceding the radiographic appearance of interstitial infiltrates. Additionally, the data suggested that corticosteroid therapy may improve survival in A–T patients with interstitial lung disease [13]. As noted earlier in this section, individuals with A–T are at increased risk for developing malignant tumors. Pulmonary parenchymal involvement secondary to lymphoma has been reported in children and young adults with A–T and can clinically and radiographically mimic ILD. Chemotherapy for malignant tumors is also a recognized risk factor for the development of pulmonary fibrosis in this population. Although lung biopsy can be performed to confirm a diagnosis of ILD in patients with A–T, the diagnostic benefits of such procedures must be carefully weighed against the risks associated with anesthesia and surgical intervention [2].

### 3.2. Bronchiectasis

Bronchiectasis is a clinic and radiological condition defined as abnormal dilatation of the bronchial wall. It arises as a consequence of pathophysiological processes involving neutrophilic inflammation, damage to the bronchial wall, loss of mucociliary clearance capacity, and airway obstruction, resulting in a vicious cycle that promotes disease progression [14,15].

Patients typically present with recurrent or persistent productive cough, progressive dyspnea, intermittent wheezing, hemoptysis, weight loss, fever, crackles, and signs and symptoms of the underlying condition responsible for the development of bronchiectasis (e.g., cystic fibrosis, infections, immunodeficiency, autoimmune diseases, genetic syndromes, tumors, and gastroesophageal reflux disease). Advanced disease may manifest through growth retardation, digital clubbing, and signs and symptoms of pulmonary hypertension [16,17,18].

Depending on etiology, bronchiectasis can be classified into two main categories: cystic fibrosis bronchiectasis (CFB) and non-cystic fibrosis bronchiectasis (NCFB) [3].

Cystic fibrosis, an autosomal recessive multisystem disorder, remains the most common cause of bronchiectasis in developed countries and should be excluded in all children presenting with bronchiectasis—particularly when it involves the upper lobes, in patients with extrapulmonary manifestations (malabsorption, pancreatitis, or intestinal obstruction), and in patients infected with *Staphylococcus aureus*, *Pseudomonas aeruginosa*, or nontuberculous mycobacteria [19,20,21].

Non-cystic fibrosis bronchiectasis (NCFB) occurs less frequently among children in developed countries, with a rate of approximately 87 cases per 1,000,000 inhabitants [19].

Globally, the prevalence of NCFB exceeds that of CFB [16]. In developing countries with low vaccination rates, infections (tuberculosis, measles, and pertussis) constitute the main etiology of NCFB, whereas in developed countries with access to advanced diagnostics, more likely causes include pulmonary aspiration (due to congenital anomalies, neuromuscular disorders, and gastroesophageal reflux), primary ciliary dyskinesia, allergic bronchopulmonary aspergillosis, and immunodeficiencies [15]. Chronic respiratory infections represent a major etiological factor for NCFB in children, including recurrent acute bronchitis (>3 episodes/year), Haemophilus influenzae infection, whooping cough, measles, tuberculosis, nontuberculous mycobacterial or atypical bacterial infection [15,22]. In a case series of 23 children (mean age of 3.6 years) with bronchiectasis, six cases (26%) occurred after pertussis or measles, all in unvaccinated children [23].

Additionally, in a series of 38 children with *Mycoplasma pneumonia*, eight developed bronchiectasis on HRCT at 1–2 years’ follow-up [24]. Bronchiectasis may also occur concomitantly with autoimmune diseases (rheumatoid arthritis, systemic lupus erythematosus, scleroderma, dermatomyositis, Sjögren syndrome, and inflammatory bowel diseases—more frequent in ulcerative colitis than Crohn’s disease) [15]. A 2024 study revealed significant genetic correlations between autoimmune diseases and bronchiectasis, particularly Crohn’s disease (r_g = 0.220, *p* = 0.037), rheumatoid arthritis (r_g = 0.210, *p* = 0.021), and ulcerative colitis (r_g = 0.247, *p* = 0.023); the same study showed rheumatoid arthritis to be an independent risk factor for bronchiectasis [25]. Genetic syndromes are rare causes of bronchiectasis. These include Marfan syndrome (connective tissue involvement), Williams–Campbell syndrome (bronchial cartilage deficiency with expiratory collapse), Mounier–Kuhn syndrome (marked dilatation of the lower trachea—wider than the vertebral column—and main bronchi with collapse), yellow nail syndrome (chronic respiratory disease, primary lymphedema, and yellow nails), and syndromes associated with immune deficiency (ataxia–telangiectasia and DiGeorge syndrome) [15,26,27,28,29].

Primary (congenital or genetic) or secondary (acquired—pharmacologic immunosuppression, HIV infection, malnutrition, or malignancies, particularly hematologic) can favor recurrent infections and thereby lead to bronchiectasis [15,20,30].

Among primary immunodeficiencies causing pediatric bronchiectasis, the most common are immunoglobulin-related defects (70–75% of cases), including X-linked agammaglobulinemia, common variable immunodeficiency, IgG subclass deficiencies, selective IgA deficiency, hyper-IgE (Job) syndrome, hyper-IgM syndrome, and Nijmegen breakage syndrome. It is therefore essential to identify primary immunodeficiencies using widely available laboratory tests: leukocyte count and serum immunoglobulin concentrations (IgG, IgA, IgM, and IgE). Prompt initiation of combined therapy with immunoglobulin replacement and antibiotics significantly reduces infection frequency and severity, lowers complication risk, improves quality of life, and enhances long-term prognosis [31].

Combined T- and B-cell immunodeficiencies (severe combined immunodeficiency, ataxia–telangiectasia/Louis–Bar syndrome, DiGeorge syndrome, and WHIM syndrome) most commonly predispose patients to infections by intracellular pathogens (recurrent pneumonia, sinusitis, and otitis media) [31]. As mentioned previously in Section 3.1, patients with ataxia–telangiectasia are predisposed to recurrent viral and bacterial infections and have an increased risk of malignancies, particularly lymphoreticular tumors (Hodgkin lymphoma, non-Hodgkin lymphoma, acute lymphoblastic leukemia, chronic lymphocytic leukemia, and multiple myeloma) [31,32,33]. Conversely, neoplasms can cause secondary immunodeficiency due to impaired synthesis of immune components, immunosuppressive treatment, and malnutrition associated with malignancy [31].

Secondary immunodeficiencies (acquired—pharmacologic immunosuppression, HIV infection, malnutrition, and malignancies, particularly hematologic) are more common than primary ones, but their association with bronchiectasis is not well understood [34]. We note that both systemic and inhaled corticosteroids may contribute to immunosuppression and increased infection risk, with reports of bronchiectasis in patients with long-standing, poorly controlled severe asthma treated with corticosteroids, and associations with tuberculosis and nontuberculous mycobacterial infections—common etiologies of bronchiectasis [35,36].

Nevertheless, over 30% of NCFB cases remain idiopathic [15].

## 4. Discussion

Ataxia–telangiectasia (A–T) is a rare multisystem autosomal recessive disorder caused by mutations in the *ATM* gene, which encodes a protein essential for the repair of DNA double-strand breaks. In addition to progressive neurological signs and pathognomonic telangiectasias, patients with A–T frequently develop pulmonary complications: recurrent respiratory infections, bronchiectases, chronic respiratory failure, and occasionally interstitial lung disease.

In this case, the diagnosis of A–T was clinically suspected on the basis of the characteristic triad (ataxia, oculomotor apraxia, and telangiectasias) and later confirmed by genetic testing (two pathogenic frameshift mutations in *ATM*).

In this patient, several structural and functional mechanisms converge to produce lung injury: repeated aspirations (documented in history and supported by barium swallow evidence of gastroesophageal reflux) due to dysphagia and impaired neuromotor coordination, commonly encountered in A–T; severe motor disorders impairing mucociliary clearance and favoring bacterial colonization; humoral immunodeficiency (low IgA and IgG) reducing mucosal anti-infective defense; poor nutritional status, compromising infection control and tissue repair; and cerebellar/brainstem hypoplasia interfering with the control of breathing, cough, and swallowing. All these elements belong to the complex clinical spectrum of A–T and act synergistically in the development of bronchiectasis.

Compared with previously reported cases of ataxia–telangiectasia, the patient in the present study shares several well-established clinical features, including recurrent sinopulmonary infections, progressive neurological impairment, and the development of bronchiectasis as a consequence of chronic respiratory disease. Similar to large cohort studies, bronchiectasis in A–T is most commonly described as a late complication of recurrent infections and impaired mucociliary clearance, often becoming clinically evident in adolescence or early adulthood. However, in contrast to typical reports in which infectious and immunodeficiency-related mechanisms predominate, the present case is distinguished by a prominent aspiration-related component secondary to severe neurodevelopmental delay and dysphagia, which appears to have played a central role in the early onset and rapid progression of structural lung disease [15,31].

This case report provides additional insight into the phenotypic variability of ataxia–telangiectasia. Although only male members were clinically affected in this family, the identification of two pathogenic heterozygous ATM variants supports the classical autosomal recessive inheritance pattern of the disease. The apparent male-limited distribution within this small pedigree is most likely explained by Mendelian segregation.

Recognizing this entity is essential, as A–T entails:

- Specific management, including immunoglobulin replacement therapy and avoidance of unnecessary radiological exposure due to radiosensitivity;
- Ongoing monitoring for pulmonary and neurological complications;
- Genetic counseling for the family, given the risk for offspring.

Limitations of the Study:

- Lack of long-term follow-up—long-term follow-up data were not available, which limits understanding of disease progression and response to treatment over time.
- Possible confusion with other diagnoses—due to overlapping features with other immunodeficiencies or neurodegenerative disorders, the diagnosis may be subject to differential diagnostic uncertainty.
- Incomplete information on extended family history—limited availability of detailed genetic or clinical information from extended family members may hinder accurate assessment of inheritance patterns.
- Lack of familial segregation analysis—genetic testing of the parents and siblings would have helped determine whether the two identified ATM variants were located in trans on opposite alleles, thereby providing stronger molecular confirmation of the autosomal recessive inheritance pattern.
- Inability to assess full neurological function at a young age—in pediatric patients, especially at a young age, neurological signs may be subtle or nonspecific, which may delay an accurate diagnosis.
- Limitations of imaging techniques—imaging findings, although suggestive, may be influenced by the quality of the technique or variability in interpretation.
- Lack of pulmonary function tests—these examinations could not be performed due to the patient’s lack of cooperation, limiting the assessment of respiratory impairment severity.
- Variable therapeutic interventions—the management strategy was individualized and may not reflect standardized treatment protocols.
- Challenges in interdisciplinary communication—occasional gaps in information exchange might have resulted in missing or incomplete clinical data during patient management.
- Low generalizability—as a single case report, the findings cannot be generalized to the wider population without further studies involving larger cohorts.

Recommendations for Future Research:

- Genetic testing and analysis—future studies should incorporate comprehensive genetic evaluation, including segregation analysis and advanced sequencing approaches, to better characterize genotype–phenotype correlations and identify genetic factors that may contribute to clinical variability in ataxia–telangiectasia.
- Long-term follow-up—prospective longitudinal studies are needed to evaluate the natural history, disease progression, and response to treatment in patients with ataxia–telangiectasia, especially those presenting primarily with respiratory symptoms.
- Larger cohort studies—studies involving larger cohorts of patients with similar clinical presentations may improve our understanding of phenotypic variability, genotype–phenotype correlations, and the spectrum of pulmonary manifestations in ataxia–telangiectasia.
- Utilization of advanced imaging technologies—more sensitive imaging methods, such as functional magnetic resonance imaging (fMRI) or high-resolution computed tomography (HRCT), should be incorporated for a more detailed assessment of pulmonary and neurological involvement.
- Comparative studies with other similar genetic disorders—clinical and genetic comparisons between ataxia–telangiectasia and other rare diseases with similar pulmonary and neurological manifestations should be performed to better understand common mechanisms and differences.
- Assessment of therapeutic interventions—the efficacy of various therapeutic strategies, including immunoglobulin replacement therapy, respiratory physiotherapy, and emerging treatments targeting genetic defects should be investigated.
- Development of diagnostic and prognostic biomarkers—research should be conducted on specific biomarkers that could facilitate early diagnosis and monitoring of disease progression.
- Multidisciplinary approach—the importance of integrated care involving immunologists, neurologists, pulmonologists, and geneticists should be emphasized to optimize diagnosis, treatment, and monitoring of patients.
- Improved communication protocols—strategies to enhance communication and data sharing among multidisciplinary teams should be explored to ensure comprehensive patient care and accurate data collection.
- Multicenter and international studies—collaboration among medical centers and institutions across different countries should be encouraged to achieve larger sample sizes and a more comprehensive understanding of the disease.

## 5. Conclusions

This case emphasizes ataxia–telangiectasia (A–T) as a rare multisystem disorder defined by progressive cerebellar ataxia and pathognomonic telangiectasias, with respiratory manifestations, such as bronchiectasis, occurring as part of its complex clinical spectrum. It highlights the importance of considering A–T in pediatric patients presenting with subtle neurological signs or immunodeficiencies, even when respiratory symptoms are notable. A comprehensive, multidisciplinary evaluation—including neurological, immunological, respiratory, and genetic assessments—is essential for accurate diagnosis. Early recognition of A–T enables not only optimized symptomatic management but also timely preventive and disease-modifying interventions, ultimately reducing morbidity and improving quality of life. This case emphasizes the importance of integrated diagnostic approaches to detect atypical or delayed presentations of A–T, ensuring prompt and coordinated care.

## Data Availability

Data is contained within the article.
